# Formulation and Evaluation of Alginate Microcapsules Containing an Uncompetitive Nanomolar Dimeric Indenoindole Inhibitor of the Human Breast Cancer Resistance Pump ABCG2 with Different Excipients

**DOI:** 10.3390/pharmaceutics17121587

**Published:** 2025-12-09

**Authors:** Krisztina Bodnár, Christelle Marminon, Florent Perret, Ádám Haimhoffer, Boglárka Papp, Pálma Fehér, Zoltán Ujhelyi, Joachim Jose, Marc Le Borgne, Ildikó Bácskay, Liza Józsa

**Affiliations:** 1 Department of Pharmaceutical Technology, Faculty of Pharmacy, University of Debrecen, 4002 Debrecen, Hungary; bodnar.krisztina@pharm.unideb.hu (K.B.); haimhoffer.adam@pharm.unideb.hu (Á.H.); papp.boglarka@pharm.unideb.hu (B.P.); feher.palma@pharm.unideb.hu (P.F.); bacskay.ildiko@pharm.unideb.hu (I.B.); 2 Doctoral School of Pharmaceutical Sciences, University of Debrecen, 4032 Debrecen, Hungary; 3 Gastroenterology and Technologies for Health Team, Centre de Recherche en Cancérologie de Lyon, Centre Léon Bérard, CNRS 5286, INSERM 1052, Université Claude Bernard Lyon 1, University of Lyon, 69373 Lyon, France; christelle.marminon@univ-lyon1.fr (C.M.); marc.le-borgne@univ-lyon1.fr (M.L.B.); 4 ENSL, CNRS, Laboratoire de Chimie, UMR 5182, 69364 Lyon, France; florent.perret@univ-lyon1.fr; 5 Department of Industrial Pharmaceutical Technology, Faculty of Pharmacy, University of Debrecen, Rex Ferenc Utca 1, 4002 Debrecen, Hungary; ujhelyi.zoltan@pharm.unideb.hu; 6 Institute of Pharmaceutical and Medicinal Chemistry, University of Münster, 48149 Münster, Germany; joachim.jose@uni-muenster.de

**Keywords:** ABCG2 inhibitor, microencapsulation, drug delivery systems, bioavailability, biocompatibility, Caco-2, MCF-7, polyvinylpyrrolidone

## Abstract

**Background/Objectives**: The ABCG2 transporter actively effluxes anticancer drugs, reducing their efficacy and promoting multidrug resistance (MDR). Developing oral formulations of poorly soluble ABCG2 inhibitors remains challenging due to their low solubility and intestinal permeability. This study aimed to formulate and evaluate an ABCG2 inhibitor using micro- and nanoscale drug delivery systems. **Methods**: To address the poor solubility and bioavailability of the corresponding active ingredient, a self-nanoemulsifying drug delivery system (SNEDDS) was developed. The SNEDDS was encapsulated into microcapsules using sodium alginate crosslinked with calcium chloride. Five microcapsule formulations were developed, varying in the inclusion of polyvinylpyrrolidone (PVP), Transcutol^®^ HP and SNEDDS. The effects of the excipients on encapsulation efficiency, swelling capacity, enzymatic stability, dissolution, cytocompatibility, and permeability were systematically evaluated. **Results**: The SNEDDS exhibited monodisperse particle sizes and efficient drug entrapment. Results revealed that formulations incorporating PVP and SNEDDS improved encapsulation efficiency and bioavailability. SNEDDS-containing formulations demonstrated superior enzymatic stability in simulated gastric and intestinal fluids and provided the highest cumulative drug release in vitro. Cytotoxicity studies conducted on Caco-2 and MCF-7 cells demonstrated that our formulations were well tolerated, indicating favorable biocompatibility. **Conclusions**: Our findings demonstrate that SNEDDS-loaded alginate microcapsules offer an efficient platform for oral delivery of dimeric ABCG2 inhibitors, combining enhanced solubility, stability, and controlled release. The optimized formulation can be regarded as a promising strategy to enhance the oral bioavailability of efflux pump inhibitors and other poorly soluble drugs.

## 1. Introduction

ATP-binding cassette (ABC) transporters are a family of membrane proteins that play a crucial role in cellular homeostasis by facilitating the transport of various substrates across biological membranes. These transporters are widely expressed in different tissues and are involved in the absorption, distribution, and excretion of endogenous and exogenous compounds, including drugs. Among them, ABCG2, also known as the breast cancer resistance protein (BCRP), has garnered significant attention due to its role in multidrug resistance (MDR) in cancer therapy [1]. ABCG2 is highly expressed in several drug-resistant cancer cells, where it actively effluxes chemotherapeutic agents, such as mitoxantrone, topotecan, and anthracyclines, thereby reducing their intracellular accumulation and limiting their cytotoxic effects. This protective function, while beneficial in physiological contexts such as the blood–brain barrier and placenta, poses a major challenge in oncology, as it diminishes the efficacy of anticancer drugs and contributes to tumor progression [1,2,3].

Efforts to overcome MDR have led to the exploration of various ABCG2 inhibitors, which can block or modulate its efflux function, thereby enhancing intracellular drug retention and improving therapeutic outcomes. Indenoindole derivatives have emerged as potent inhibitors of the ABCG2 receptor, demonstrating significant promise as molecular scaffolds for the rational design of more effective and selective modulators. These compounds possess a rigid polycyclic aromatic framework that enables favorable π–π stacking and hydrophobic interactions within the ABCG2 binding pocket. Structural modifications on the indenoindole core have been shown to strongly influence inhibitory potency and selectivity, providing valuable insights into the structure–activity relationship of this compound class. Therefore, indenoindole-based molecules represent an attractive platform for the development of next-generation ABCG2 inhibitors with improved pharmacokinetic and pharmacodynamic profiles [4,5,6,7].

However, many of these inhibitors suffer from poor aqueous solubility, low bioavailability, and potential off-target toxicity, limiting their clinical applicability [8,9]. To address these challenges, advanced drug delivery systems, particularly those at the micro- and nanoscale, have emerged as promising approaches. These systems can improve the solubility, stability, and controlled release of therapeutic agents while enhancing their targeted delivery to cancer cells [10,11].

SNEDDS have gained increasing interest in pharmaceutical research to improve the solubility and bioavailability of poor water-soluble compounds. SNEDDS are isotropic mixtures of oils, surfactants, and cosurfactants that spontaneously form fine oil-in-water nanoemulsions upon contact with gastrointestinal fluids, enhancing drug dissolution and absorption [12,13]. Despite their advantages, liquid SNEDDS formulations may suffer from stability issues, necessitating their conversion into solid dosage forms for better handling and controlled drug release. One effective approach is the encapsulation of SNEDDS into biocompatible polymeric microcapsules, which can further improve drug stability, protect against enzymatic degradation, and provide sustained release profiles [14].

Excipients such as Transcutol^®^ HP, Labrasol^®^, Capryol^®^ 90, and polyvinylpyrrolidone (PVP) have gained considerable attention for their ability to enhance the solubility, stability, and bioavailability of poorly water-soluble drugs. Transcutol^®^ HP (diethylene glycol monoethyl ether) is a well-known solubilizer and penetration enhancer that improves drug diffusion across biological membranes [15]. Labrasol^®^ (caprylocaproyl macrogol-8 glycerides) and Capryol^®^ 90 (propylene glycol monocaprylate (type II)) are lipid-based excipients commonly employed in self-emulsifying and nano/microemulsion systems, where they facilitate lipid solubilization and promote efficient drug absorption through the gastrointestinal tract [16,17]. PVP is a widely used hydrophilic polymer in microencapsulation due to its excellent film-forming ability, biocompatibility, and capacity to enhance the solubility of poorly water-soluble drugs. In microcapsule formulations, PVP can act as a stabilizing and matrix-forming agent, contributing to the formation of uniform and mechanically stable shells around the encapsulated core [18]. Its strong hydrogen-bonding potential allows favorable interactions with both hydrophilic and lipophilic drug molecules, thereby improving encapsulation efficiency and controlling drug release profiles. Moreover, PVP can prevent drug crystallization during microcapsule drying and storage, ensuring a more stable amorphous state that enhances dissolution and bioavailability. The polymer’s molecular weight and concentration can be optimized to tailor the release kinetics and degradation behavior of the microcapsules, making PVP a versatile excipient in the design of sustained and controlled drug delivery systems [19,20]. Together, these excipients offer complementary functionalities that can be strategically combined to develop advanced drug delivery systems with optimized solubility and controlled-release characteristics.

Alginate, a natural polysaccharide widely used in drug delivery, is particularly suitable for microencapsulation due to its non-toxic, biodegradable, and mucoadhesive properties [21,22,23]. In addition, they offer efficient targeting, sustained delivery, and controllable release profiles. During the preparation of alginate-based microbeads, the active substance is entrapped within an alginate gel matrix, which is formed through ionic crosslinking between the uronic acid residues of alginate and divalent cations such as Ca^2+^, Ba^2+^, or Sr^2+^ [24,25,26].

In this study, we aimed to develop and characterize an innovative drug delivery system for an ABCG2 inhibitor by incorporating it into SNEDDS and further encapsulating this formulation into alginate-based microcapsules. The model compound used in this study was a dimeric indenoindole-based ABCG2 inhibitor synthesized and previously published by the Cancer Research Center of Lyon (CRCL) [6]. We systematically investigated the physicochemical properties of the developed formulations, including particle size, surface morphology, encapsulation efficiency, and swelling behavior. Additionally, we evaluated the cytocompatibility of microcapsules using Caco-2 and MCF-7 cell lines to assess their safety for potential biomedical applications. By enhancing the bioavailability and stability of ABCG2 inhibitors, this research contributes to the ongoing efforts to develop more effective strategies for combating MDR in breast cancer and other chemoresistant malignancies.

## 2. Materials and Methods

### 2.1. Materials

The active pharmaceutical ingredient (API) selected for this study, ABCG2 inhibitor **7b**, was synthesized by the CRCL research laboratory [6]. The SNEDDS ingredients—Labrasol^®^ (caprylocaproyl macrogol-8 glycerides), Transcutol^®^ HP (diethylene glycol monoethyl ether) and Capryol^®^ 90 (propylene glycol monocaprylate (type II))—were purchased from Gattefossé (Lyon, France). Low viscosity grade sodium alginate was sourced from BÜCHI Labortechnik AG (Flawil, Switzerland). The human adenocarcinoma cancer cell line (Caco-2) and the human breast cancer cell line (MCF-7) were acquired from the European Collection of Authenticated Cell Cultures (ECACC, Public Health England, Salisbury, UK). Culturing flasks and 96-well cell culture plates were supplied by VWR International (Debrecen, Hungary). Transwell^®^ 24-well cell culture inserts were obtained from Greiner Bio-One Hungary Kft. (Mosonmagyarovar, Hungary). All other reagents and chemicals were procured from Sigma-Aldrich (Budapest, Hungary).

### 2.2. Characterization of the Inhibitor Molecule

The physicochemical characterization of the ABCG2 inhibitor **7b** (referred as API, C_52_H_44_N_2_O_6_, 792.92 g/mol) was carried out using the SwissADME web tool (https://www.swissadme.ch). Parameters related to lipophilicity, molecular size, polarity, insolubility, degree of unsaturation, and molecular flexibility were determined. Furthermore, water solubility, pharmacokinetics, and bioavailability were also assessed during the SwissADME analysis [27].

### 2.3. Solubility Studies

The purpose of the conducted solubility studies was to identify appropriate SNEDDS components capable of effectively solubilizing an ABCG2 transporter inhibitor molecule. To accomplish this, 100 mg of the ABCG2 inhibitor was added to 1 mL of surfactant (Labrasol^®^), co-surfactant (Transcutol^®^ HP), and to the oil phase (Capryol^®^ 90) of the SNEDDS formulation. The mixture was vortexed thoroughly and allowed to equilibrate at room temperature (24.0 °C ± 0.5 °C) for 48 h. Following equilibration, saturation solubility was assessed [28]. To remove the undissolved ABCG2 inhibitor **7b**, the samples were centrifuged at 10,000 rpm for 10 min. The supernatant was then filtered through a 0.45 µm membrane filter and diluted with absolute ethanol. The concentration of the dissolved ABCG2 inhibitor was measured spectrophotometrically at 415 nm.

### 2.4. Determination of the Emulsification Efficiency

Labrasol^®^ and Transcutol^®^ HP were evaluated for their ability to emulsify the oil phase. To assess the emulsification efficiency 1 mL of each surfactant was mixed with 1 mL of Capryol^®^ 90. The resulting mixture was vortexed and heated at 60 °C for 5 min to achieve homogenization. Afterward, 500 μL of the mixture was diluted to 50 mL with distilled water. The emulsions were left to stand for 2 h, and their transmittance was measured using a UV–VIS spectrophotometer at 650 nm [28]. The percentage transmittance of each emulsion was calculated in triplicate, and the average values ± SD were determined.

### 2.5. Formulation and Investigation of Self-Nanoemulsifying Drug Delivery Systems

Self-nanoemulsifying system was prepared by mixing Labrasol^®^, Transcutol^®^ HP and Capryol^®^ 90 in 1:1:1 ratio. The mixture was then heated to 37 °C and blended using a Schott Tritronic dispenser (SI Analytical, Mainz, Germany) in conjunction with a Radelkis magnetic stirrer (Radelkis, Budapest, Hungary, version number: OP-912). ABCG2 inhibitor **7b** (10 mg/mL) was dissolved in the mixture at room temperature under continuous agitation.

In our preliminary solubility and emulsification screening studies, several ratios of Labrasol^®^, Transcutol^®^ HP and Capryol^®^ 90 were tested to identify mixtures providing the optimal balance between drug solubilization capacity, self-emulsification efficiency and nanoemulsion stability. The 1:1:1 ratio consistently yielded the smallest droplet sizes, rapid emulsification upon dilution, and the highest solubility of compound **7b** compared with the other tested compositions. This ratio is further supported by our previous study showing that ternary systems composed of a hydrophilic surfactant, a co-surfactant and a medium-chain lipid in equal proportions efficiently form stable nanoemulsions with narrow size distribution for poorly soluble drugs [29].

The Zetasizer Nano S device (Malvern Panalytical, Malvern, UK, serial number: MAL1226409) was used to determine droplet size, zeta potential, and polydispersity, as these data are essential for characterizing self-emulsifying systems. For the analysis, 100 μL of the self-emulsifying system was dissolved in 1 mL of distilled water. The sample was analyzed in triplicate in all measurements.

To evaluate the drug loading efficiency, 10 mg of the formulated systems were diluted with 100 mL of absolute ethanol. The mixture was centrifuged in an ultracentrifuge tube equipped with a filter membrane, and the drug content of the filtrate was measured spectrophotometrically at 415 nm. The efficiency was calculated using the following equation [29] (Equation (1)):

(1) $$Drug\;loading\;efficiency(\%)=\frac{Amount\;of\;API\;measured\;in\;10\;mg\;SNEDDS}{Amount\;of\;API\;added}\times 100$$

### 2.6. Formulation of Alginate Microcapsules

#### 2.6.1. Preparation of Sodium-Alginate and Calcium Chloride Dihydrate Solutions

A 1.5% (*w*/*v*) polymer solution was prepared by dissolving 8.25 g of low-viscosity sodium alginate in 500 mL of distilled water. The mixture was stirred continuously at 300 rpm for 4 h at room temperature (24 °C) to ensure homogeneity. To prepare a 100 mM calcium chloride dihydrate solution, 7.35 g of calcium chloride dihydrate was dissolved in 500 mL of distilled water [30].

#### 2.6.2. Preparation of ABCG2 Inhibitor-Loaded Alginate Microcapsules

Four different formulations containing ABCG2 inhibitor **7b** (1% *w*/*v*) were developed, and microcapsules without the active ingredient (MC0) were also formulated (Table 1). During the formulation of microcapsules, Transcutol^®^ HP and polyvinylpyrrolidone (PVP) were used as solubilizing excipients. In the first composition (MC1), the active ingredient was a solid dispersion prepared by co-grinding the ABCG2 inhibitor **7b** with PVP. Drug and polymer were weighed in a 1:2 *w*/*w* ratio and ground in a mortar with a pestle for 45 min, ensuring uniform mixing. The powder was sieved (250 µm) and stored in a desiccator. This simple approach aims to reduce particle size and disperse the drug within the polymeric matrix, improving wettability and dissolution. In the second it was dissolved in Transcutol^®^ HP, while in the third (MC3), a mixture of Transcutol^®^ HP and PVP was used. The fourth formulation involved the microencapsulation of SNEDDS itself (MC4), whereas in the fifth, PVP was also added (MC5).

A 1.5% aqueous sodium alginate solution was used for the preparation of the microcapsules, in which the previously mentioned compositions were dispersed. In all cases, the inhibitor concentration was 1% *w*/*v*. The polymer-drug mixtures were loaded into a syringe and processed using the BÜCHI Encapsulator B-395 Pro apparat. The solutions passed through an electric field generated between the nozzle with a diameter of 300 µm. A 1000 V electrode separated the solution into uniformly sized droplets at a frequency of 1400 Hz. The alginate beads were then left to harden in a calcium chloride solution for 10 min. The resulting fine particles were washed with the hardening solution, filtered through a 0.45 µm pore-size membrane using a vacuum pump, and freeze-dried for 24 h at −110 °C [31].

### 2.7. Encapsulation Efficacy

To assess the drug content encapsulated within the microcapsules, a 1 mL sample was taken from the 100 mM calcium chloride hardening solution immediately after the formulation of microcapsules. The drug concentration was analyzed by UV-VIS spectrophotometer (415 nm). The encapsulation efficiency (EE) was determined using the following equation:

(2) $$EE(\%)=\left(\frac{total\;drug\;amount-free\;drug\;amount}{total\;drug\;amount}\right)\times 100$$

where total drug amount was referred to the initial amount of drug used in the formulation, while free drug amount was referred to the drug that was not encapsulated and remains in the hardening solution [31,32].

### 2.8. Swelling Behavior

The swelling behavior of the formulated microcapsules was assessed gravimetrically. Initially, 100 mg of dry microcapsules were weighed and immersed in 100 mL of distilled water at room temperature. After 1 h, the beads were removed, and any excess moisture was removed via vacuum filtration. The equilibrium water uptake (EWU) was then calculated using the following equation:

(3) $$EWU=\left(\frac{Ws-Wd}{Ws}\right)\times 100$$

where Ws represents the weight of the hydrated particles and Wd is the initial dry weight [31].

### 2.9. Scanning Electron Microscopy

Morphological characterization was performed using a Thermo Scientific™ Axia™ ChemiSEM™ Scanning Electron Microscope (Auro-Science Consulting, Budapest, Hungary). Samples were mounted onto fixtures using double-sided graphite adhesive tape, and any excess graphite was removed by argon gas flushing. No surface pre-treatments or post-measurement corrections were applied during the analysis. SEM imaging was conducted under high vacuum at an accelerating voltage of 30 kV and a dwell time of 15 µs. All images were acquired at 1000× magnification [30].

### 2.10. In Vitro Dissolution Study

To investigate the in vitro dissolution profile of the formulated microbeads containing the ABCG2 transporter-inhibiting compound, a USP-compliant paddle apparatus (Erweka DT 800, Erweka GmbH, Langen, Germany) was used. Dissolution studies were performed at a paddle rotation speed of 100 rpm in 100 mL of freshly prepared simulated intestinal fluid (SIF) without pancreatin (pH 6.8), maintained at 37 °C [29,33].

For each experiment, 100 mg of dried microbeads were added to the dissolution vessel. Aliquots of 1000 µL were withdrawn from the dissolution medium at predefined time points for analysis: 0, 4, 8, 12 and 24 h. The samples were filtered through 0.45 µm membrane filters prior to quantification. The amount of released active substance was determined spectrophotometrically at 415 nm using a previously established calibration curve.

### 2.11. Enzymatic Stability

To assess the enzymatic stability of the microbeads, degradation experiments were performed in simulated gastric fluid (SGF) containing pepsin and simulated intestinal fluid (SIF) containing pancreatin, both prepared in accordance with the European Pharmacopoeia guidelines [33,34]. Pepsin exhibited an activity of 400 units/mg, while pancreatin showed a protease activity of about 200 USP units/mg. Microcapsules were incubated in 100 mL of SGF for 2 h, followed by incubation in SIF for an additional 4 h. All experiments were conducted at 37 °C with continuous agitation at 100 rpm. At pre-determined intervals, 1 mL aliquots were collected and immediately mixed with an equal volume of ice-cold quenching reagent—0.10 M NaOH for SGF samples or 0.10 M HCl for SIF samples—to terminate enzymatic activity. The remaining drug content was then quantified spectrophotometrically at 415 nm.

### 2.12. Transepithelial Electrical Resistance (TEER) Measurements

Transepithelial electrical resistance (TEER) measurements were conducted to evaluate the integrity of epithelial barriers formed by Caco-2 and MCF-7 cell monolayers. Cells were seeded at a density of 4 × 10^4^ cells per well and cultured until tight monolayers were established. The electrical resistance was determined using a Millicell-ERS system (Millipore, Merck, Waltham, MA, USA), and only those monolayers with TEER values ranging from 800 to 1000 Ω·cm^2^ for the Caco-2 cells and 60 to 100 Ω·cm^2^ for the MCF-7 cells were selected for subsequent assessments. MCF-7 cells are relatively leaky and less tight monolayer characteristics compared to Caco-2 intestinal cells which have TEER values in the thousands Ω·cm^2^ range [35].

Test solutions were prepared by dispersing microcapsules in PBS at 1% (*w*/*v*). Each suspension was homogenized via vortexing and sonication to ensure uniformity. During the assay, cell monolayers were treated with the test solutions, and TEER values were continuously monitored for one hour to assess the immediate effects on barrier integrity. Thereafter, TEER values were recorded at 4, 8, and 12 h. To evaluate the recovery of barrier function, measurements were extended for an additional 24 h after treatment, allowing the assessment of tight junction restoration.

PBS (phosphate-buffered saline) and 10% (*w*/*v*) Triton X-100 served as controls for barrier preservation and disruption, respectively.

### 2.13. In Vitro Permeability Assay

Permeability studies were conducted using both Caco-2 and MCF-7 cell monolayers cultured on Transwell^®^ inserts (24-well format, polycarbonate membrane, 1.12 cm^2^ surface area, 0.4 µm pore size). Cells were seeded onto each insert at a density of 4 × 10^4^ cells, and monolayer integrity was verified by transepithelial electrical resistance (TEER) measurements prior to the experiments.

The same test formulations as used for TEER analysis (1% (*w*/*v*) in PBS) were applied. Dispersions were thoroughly mixed before application. For each assay, 400 µL of the formulation was added to the apical side of each insert, while 1400 µL of culture medium was added to the basolateral chamber. Samples of 50 µL were collected from the basolateral side at 4, 8, 12 and 24 h to monitor compound permeation [30].

Collected samples were analyzed by UV-Vis spectrophotometry at 415 nm to determine the amount of inhibitor molecules permeated through the cell monolayers.

### 2.14. MTT Viability Assay

For cell viability assays, different concentrations (0.01, 0.05, 0.10, 0.50 and 1% *w*/*v*) of samples were prepared in sterile PBS. The cytotoxicity of the excipients used for the formulation, the free ABCG2 inhibitor molecule, and the microcapsules were also evaluated on Caco-2 and MCF-7 cell lines.

The cytotoxic effects of these formulations were assessed using the MTT assay [36]. Caco-2 and MCF-7 cells were seeded in 96-well plates (VWR International Inc., Debrecen, Hungary) at a density of 10^4^ cells per well. After 5 days of culture, the medium was removed, and cells were treated with 100 µL of the test solutions for 2 h at 37 °C. Subsequently, the solutions were removed and 0.5 mg/mL MTT solution (dissolved in PBS) was added to each well. After a 3 h incubation at 37 °C, the MTT dye was removed and 0.1 mL of an isopropanol-1 M hydrochloric acid (25:1) solution was added to solubilize the formed formazan crystals. Absorbance was measured at 565 nm with a 690 nm reference using a Thermo-Fisher Multiskan Go microplate reader (Thermo-Fisher, Waltham, MA, USA, Cat. No. N10588). PBS and 10% (*w*/*v*) Triton X-100 served as negative and positive controls, respectively. Cell viability was expressed as a percentage relative to untreated control cells incubated with PBS for 24 h.

### 2.15. Statistical Analysis

Data processing and statistical analyses were performed using Microsoft Excel 2016 (version 16.0.10827.20118) and GraphPad Prism (version 10.6.1; GraphPad Software, San Diego, CA, USA). Results are presented as means ± standard deviation (SD). Multiple group comparisons were conducted using one-way or two-way ANOVA followed by either Dunnett’s multiple comparison test or Tukey’s multiple comparison test, depending on whether the comparisons were made against a single control group or among all groups, respectively [29]. Statistically significant differences are indicated by asterisks in the figures, with significance defined as *p* < 0.05.

## 3. Results

### 3.1. In Silico Characterization of the ABCG2 Inhibitor ***7b***

**Figure 1:**
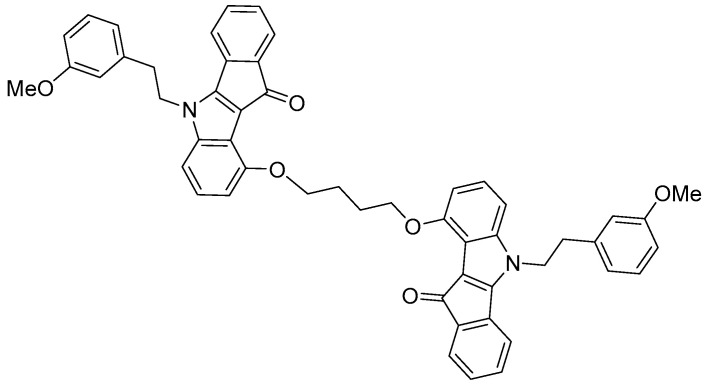
The molecular structure of the ABCG2 inhibitor **7b** used in the present study.

The molecule **7b** is a dimeric indeno[1,2-*b*]indole-based compound specifically designed as a highly potent uncompetitive inhibitor of the ABCG2 transporter (*K*i = 17 nM, Figure 1) [6]. Its complex structure features two methoxy-substituted aromatic systems connected through a flexible ether linkage, which facilitate optimal binding within the ABCG2 substrate pocket. The presence of large aromatic rings and methoxy groups enhances both lipophilicity and molecular rigidity, characteristics that are favorable for strong transporter inhibition. These structural features contribute to the high affinity and selectivity of inhibitor **7b** for ABCG2, making it a promising candidate for overcoming multidrug resistance in cancer therapy [6].

**Figure 2:**
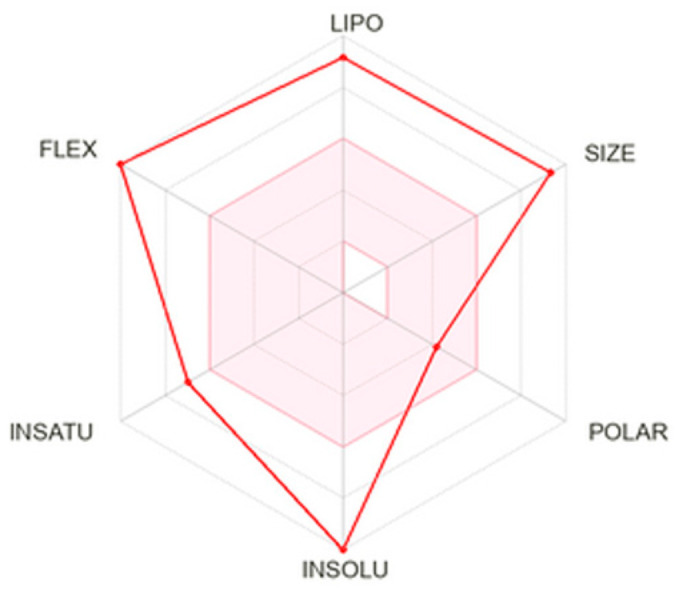
SwissADME radar chart of the physicochemical properties of the ABCG2 inhibitor **7b**. The axes represent critical factors influencing drug-likeness and bioavailability: lipophilicity (LIPO), molecular size (SIZE), polarity (POLAR), insolubility (INSOLU), degree of unsaturation (INSATU), and molecular flexibility (FLEX). The red polygon shows how the compound scores along each parameter relative to the optimal range for oral drug candidates.

According to the analysis, the compound **7b** exhibited high lipophilicity and molecular size, as well as considerable conformational flexibility (Figure 2). The Log P_o/w_ value of **7b** was 6.71, indicating the compound’s lipophilic character. **7b** also showed good membrane permeability but potentially limited aqueous solubility, which could affect its absorption and formulation.

### 3.2. Characterization of the Formulated Self-Emulsifying System

#### 3.2.1. Emulsification Efficiency of the Surfactant and Co-Surfactant

To determine the emulsification efficiency of Labrasol^®^ and Transcutol^®^ HP the percentage transmittance values of the dispersions were calculated. The emulsification efficiency of Labrasol^®^ and Transcutol^®^ HP was assessed both individually and in combination. When applied alone, Labrasol^®^ exhibited a transmittance value of 81.02% ± 0.48%, while Transcutol^®^ HP showed a value of 77.15% ± 0.24%. Notably, when the two excipients were combined, the transmittance exceeded 90% (90.54 ± 0.84%), indicating a synergistic effect and enhanced emulsification efficiency compared with the individual components.

#### 3.2.2. Solubility Studies

**Figure 3:**
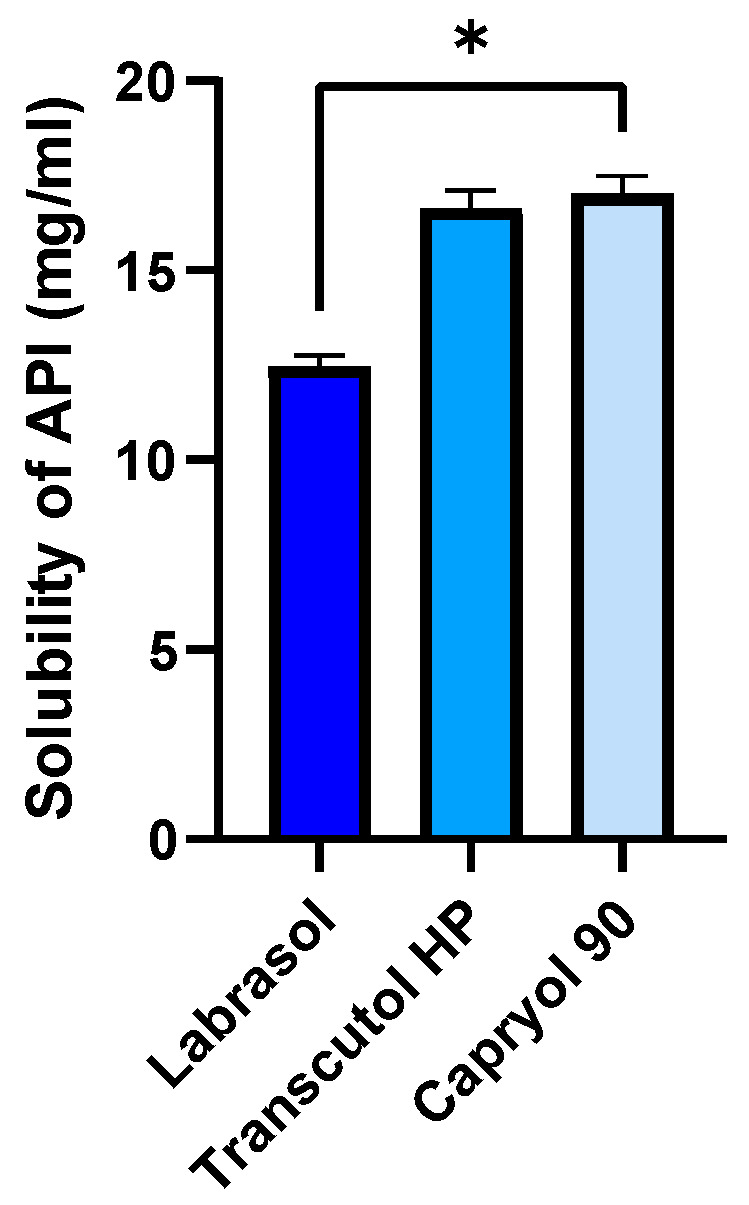
Solubility of the ABCG2 inhibitor **7b** (referred as API) in SNEDDS components at room temperature. An ordinary one-way ANOVA test and Tukey’s multiple comparison tests were performed to compare the solubilizing activity of the components. Statistically significant differences are indicated by * for *p* < 0.05.

The solubility of ABCG2 inhibitor **7b** in the individual SNEDDS components at room temperature is shown in Figure 3. Among the tested excipients, Transcutol^®^ HP and Capryol^®^ 90 exhibited the highest solubilizing capacity, both exceeding 17 mg/mL. In contrast, Labrasol^®^ resulted in a markedly lower solubility of approximately 13 mg/mL. Statistical analysis using one-way ANOVA followed by Tukey’s multiple comparison test revealed a significant difference between Labrasol^®^ and the other two components (*p* < 0.05). These findings indicate that Transcutol^®^ HP and Capryol^®^ 90 are more efficient in solubilizing the inhibitor molecule, suggesting their potential as key components in the formulation to achieve high drug loading and improve bioavailability.

#### 3.2.3. Droplet Size, Polydispersity Index, Zeta Potential and Drug Loading Efficiency

The droplet size, polydispersity index (PDI), zeta potential, and drug loading efficiency of the SNEDDS formulations are summarized in Table 2. The blank SNEDDS showed a mean droplet size of 45.31 ± 1.73 nm with a narrow size distribution (PDI = 0.189 ± 0.009), indicating the formation of a uniform nanoemulsion. Upon incorporation of the API, a significant increase in droplet size to 124.87 ± 12.45 nm was observed, while the PDI remained low (0.195 ± 0.011), suggesting that the system maintained good homogeneity despite the increased size. The zeta potential values were highly negative (−34.23 ± 1.88 mV for the blank and −32.90 ± 2.01 mV for the API-loaded SNEDDS), which indicates good colloidal stability. The drug loading efficiency was high, with a mean value of 98.07 ± 4.15%, demonstrating the excellent solubilization capacity of the selected formulation components.

### 3.3. Morphological Characterization by Scanning Electron Microscopy (SEM)

**Figure 4:**
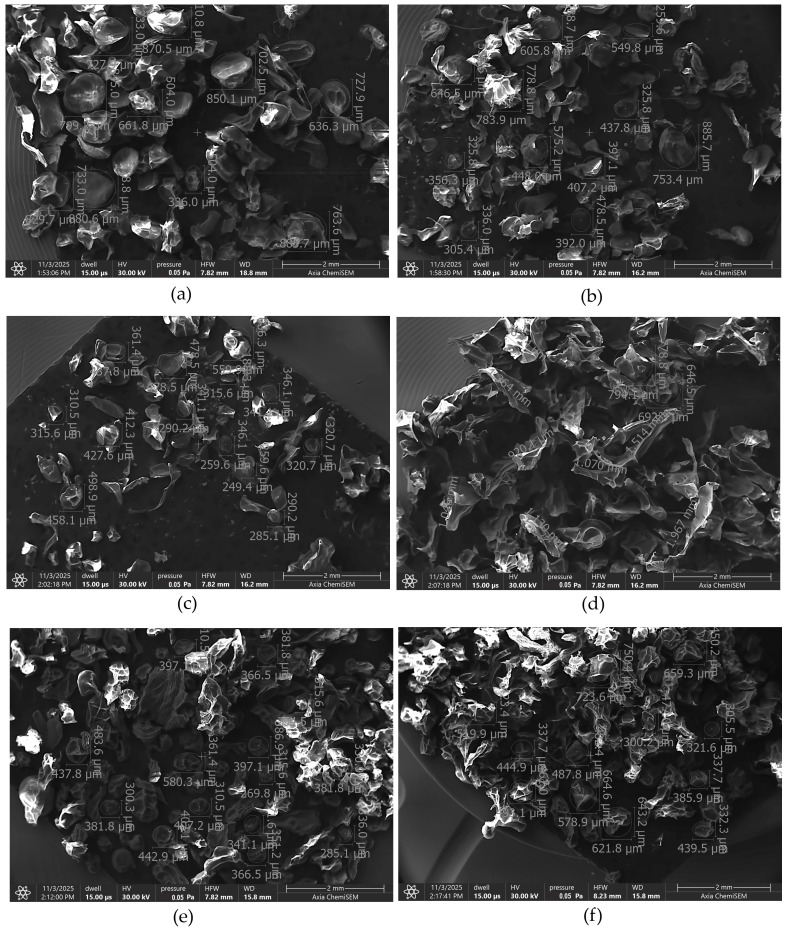
SEM image of empty microcapsules (**a**), microcapsules containing ABCG2 inhibitor **7b** (**b**), ABCG2 inhibitor **7b** and PVP (**c**), Transcutol^®^ HP (**d**), SNEDDS (**e**) and SNEDDS and PVP (**f**).

The surface morphology and particle size distribution of the six microcapsule formulations were examined by SEM (Figure 4). Distinct morphological differences were observed depending on the composition of the formulation.

The empty microcapsules (Figure 4a) displayed a broad size distribution ranging approximately from 330 µm to 850 µm. Incorporation of the ABCG2 inhibitor **7b** (Figure 4b) maintained a comparable size range (300–780 µm), while the addition of PVP (Figure 4c) led to a more uniform and less porous appearance, with smaller mean particle diameters (250–460 µm) and denser morphology. This change can be attributed to the film-forming properties of PVP, which promote better crosslinking and surface stabilization during drying.

Formulations containing Transcutol^®^ HP (Figure 4d) exhibited a markedly different microstructure with fused surface regions and larger average diameter (650–1070 µm). The presence of the glycol-based co-solvent likely increased droplet coalescence and particle aggregation, producing a denser but less spherical morphology.

The SNEDDS-loaded microcapsules (Figure 4e) showed a more homogeneous and compact morphology, with mean sizes around 370–580 µm, while the SNEDDS + PVP formulation (Figure 4f) showed a size range between 440 and 725 µm. The presence of nonionic surfactants in the SNEDDS and Transcutol^®^ HP-containing formulations increased the surface roughness and reduced sphericity, reflecting changes in interfacial tension during droplet formation and solvent evaporation. These observations indicate that the inclusion of surfactants and co-solvents affects the rheology and interfacial properties of the alginate dispersion.

### 3.4. Encapsulation Efficiency

The encapsulation efficiency of the different formulations increased progressively from MC1 to MC5 (Table 3), ranging from 61.21 ± 3.88% to 95.66 ± 2.86%. This trend reflects the impact of formulation components on drug entrapment. MC1, containing only sodium alginate, PVP and the ABCG2 inhibitor **7b**, showed the lowest EE, indicating limited drug retention in the polymer matrix. The addition of Transcutol^®^ HP in MC2 and MC3 resulted in a marked increase in EE, suggesting improved solubilization and distribution of the inhibitor within the microcapsules. Further incorporation of PVP and SNEDDS led to a substantial enhancement of encapsulation efficiency, with MC5 reaching the highest value (>95%). These findings highlight the synergistic effect of solubilizing excipients and polymeric additives in promoting efficient drug entrapment within the microcapsules.

### 3.5. Swelling Behavior

**Figure 5:**
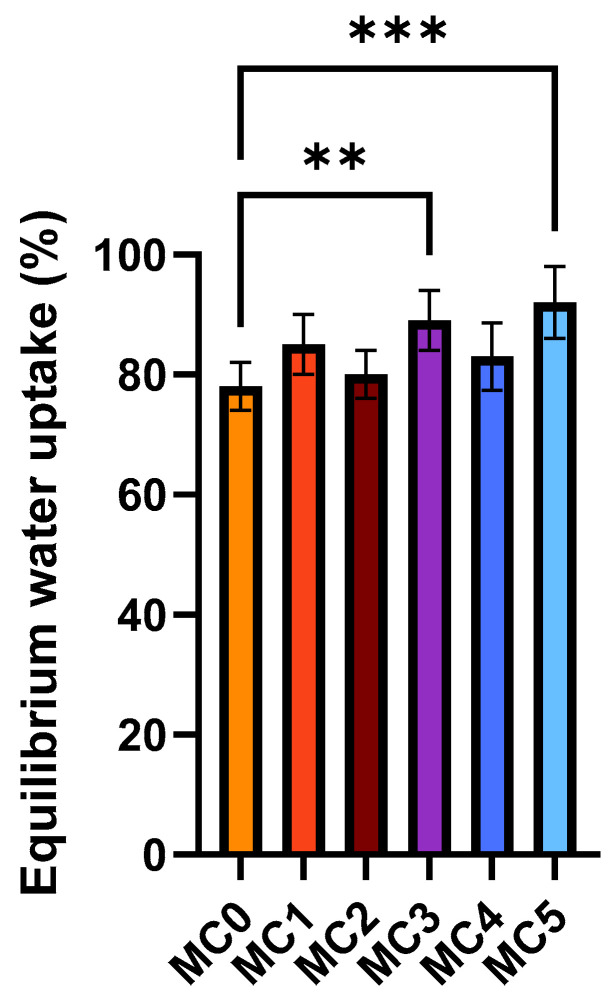
Swelling behavior of the microcapsules in distilled water. Data represent equilibrium water uptake for each composition (mean ± SD, *n* = 6). Statistical analysis was performed using one-way ANOVA with Dunnett’s multiple comparison test to compare the different formulations with the empty microcapsule (MC0). Statistically significant differences are indicated by ** (*p* < 0.01) and *** (*p* < 0.001).

Figure 5 presents the equilibrium water uptake of the microcapsules in distilled water (mean ± SD, *n* = 6). The empty alginate microcapsule (MC0) showed the lowest equilibrium water uptake, whereas formulations containing additional excipients exhibited enhanced swelling capacity. The presence of hydrophilic additives, such as PVP in MC1, MC3 and MC5, increased water absorption compared with MC0. Similarly, the incorporation of SNEDDS (MC4 and MC5) further elevated equilibrium water uptake. These results indicate that the inclusion of amphiphilic and polymeric components promotes water diffusion and retention within the alginate matrix, resulting in higher swelling degrees compared with the control formulation. In alginate-based systems, a greater degree of swelling increases the water content and porosity of the polymer matrix, which enhances the diffusion of encapsulated molecules through the network. This facilitates more controlled and sustained release of active substances, an important property for oral drug delivery systems. Additionally, better swelling behavior indicates an optimized polymer cross-linking density that balances structural stability with flexibility, promoting both mechanical integrity and desirable release kinetics [37].

### 3.6. Enzymatic Stability Assay

**Figure 6:**
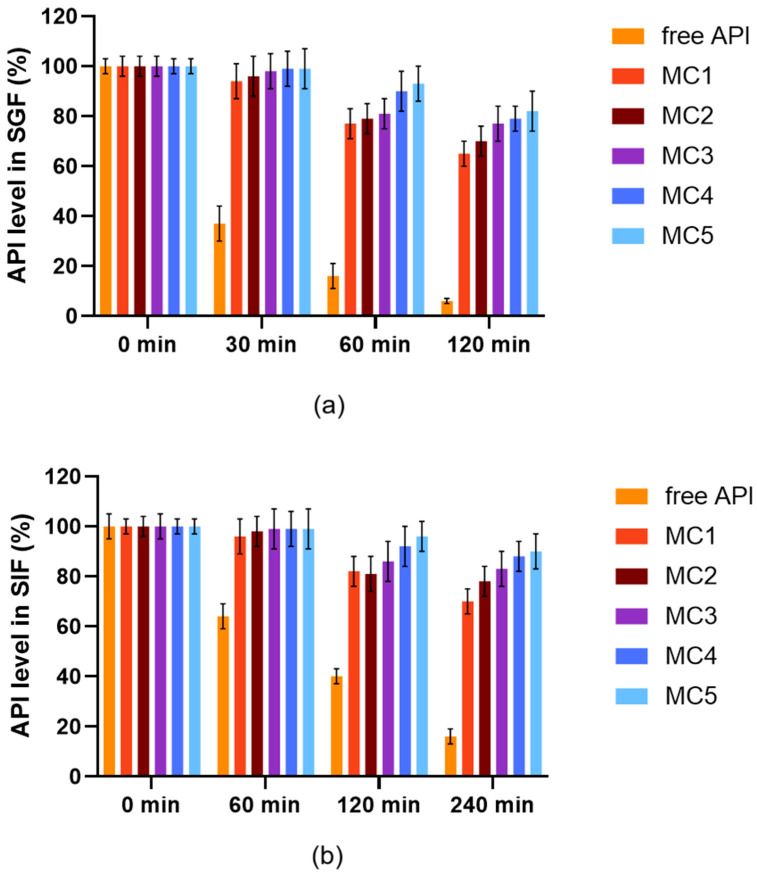
Enzymatic stability of microcapsules and free ABCG2 inhibitor **7b** (referred as API) in SGF (**a**) and in SIF medium (**b**) at 37 °C with continuous agitation at 100 rpm. Each data point represents the mean ± SD; *n* = 6. Data were analyzed by one-way ANOVA at each time point, followed by Dunnett’s multiple comparisons test versus the free API. Except for time 0, all time points showed a statistically significant difference between the free API and MC1–MC5 (*p* < 0.0001).

Figure 6 represents the enzymatic stability of the free ABCG2 inhibitor and encapsulated formulations in simulated gastric fluid (Figure 6a) and simulated intestinal fluid (Figure 6b) at 37 °C with continuous agitation (100 rpm). The ABCG2 inhibitor **7b** content was quantified over time to assess degradation and release behavior (mean ± SD, *n* = 6). In both media, the free inhibitor molecule exhibited rapid degradation, with a substantial decrease in API levels within 30–60 min in SGF and 60–120 min in SIF. In contrast, all microcapsule formulations (MC1–MC5) maintained significantly higher API stability throughout the incubation period. Among encapsulated samples, MC4 and MC5 demonstrated the highest retention of API content in both gastric and intestinal environments over the course of the experiment. This superior effect can be attributed to the SNEDDS incorporated in these formulations, which encapsulated the ABCG2 inhibitor within a nanoemulsion, affording enhanced protection against enzymatic degradation. These findings indicate that the SNEDDS-based microcapsules (MC4 and MC5) are particularly effective for stabilizing the ABCG2 inhibitor **7b**, potentially improving its oral bioavailability by preventing premature loss in the gastrointestinal tract.

### 3.7. In Vitro Dissolution Study

**Figure 7:**
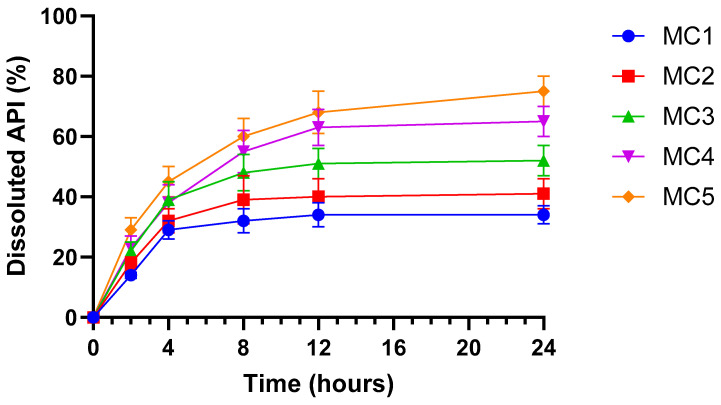
In vitro dissolution profile of ABCG2 inhibitor **7b** (referred as API) from microbeads in simulated intestinal fluid (SIF) without pancreatin (pH = 6.8). Each data point represents the mean ± SD; *n* = 6. Data were analyzed by one-way ANOVA at the final time point, followed by Dunnett’s multiple comparisons test versus MC1. Significant differences (*p* < 0.001) were found for MC3, MC4 and MC5.

Figure 7 illustrates the dissolution profile of the ABCG2 inhibitor **7b** released from the different microcapsule formulations in simulated intestinal fluid (SIF) without pancreatin (pH 6.8) over 24 h (mean ± SD, *n* = 6). All microcapsules demonstrated a sustained release pattern, with a gradual increase in dissolved API over time. Among the formulations, MC5 exhibited the fastest and highest cumulative dissolution, followed by MC4 and MC3, indicating enhanced release kinetics. In contrast, MC1 and MC2 showed slower and lower overall API release. The improved dissolution observed in MC5 and MC4 can be linked to their inclusion of SNEDDS, which enhances the solubilization and dispersion of the ABCG2 inhibitor within the dissolution medium. These data suggest that SNEDDS-containing microcapsules facilitate more efficient drug release under intestinal conditions, potentially improving drug bioavailability.

### 3.8. Screening of Cytotoxicity

**Figure 8:**
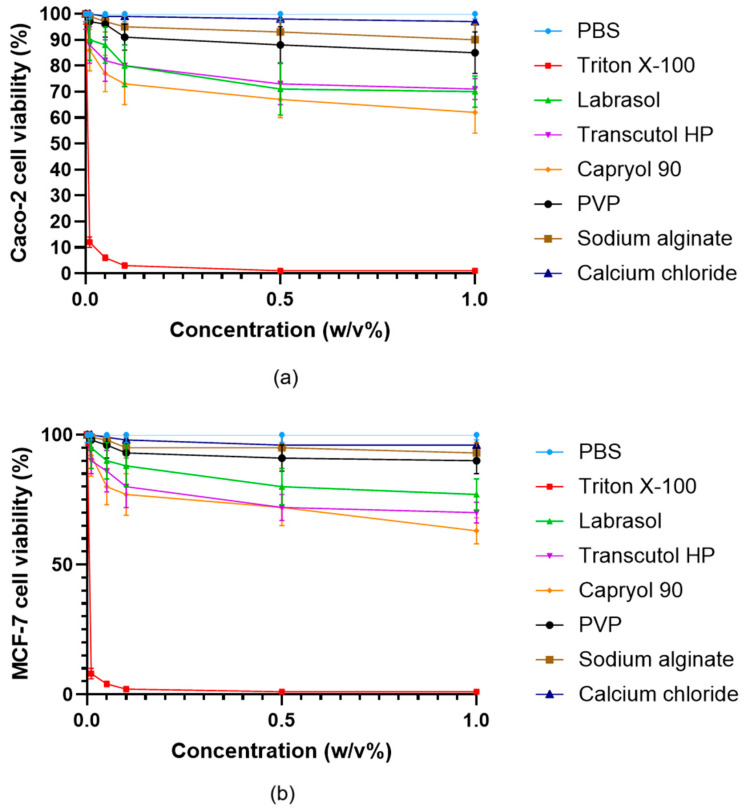
Viability of Caco-2 (**a**) and MCF-7 (**b**) cells incubated with the excipients used during the formulation of microcapsules. Cell viability is expressed as a percentage of the negative control (PBS). Data are presented as mean ± SD; *n* = 6. Two-way ANOVA followed by Dunnett’s test vs. PBS was performed to evaluate the effects of the excipients on cell viability. Significant differences (*p* < 0.001) were found for Triton X-100, Labrasol^®^, Transcutol HP^®^, Capryol 90^®^, while PVP, sodium alginate and calcium chloride showed no significant cytotoxic effects in either Caco-2 or MCF-7 cells.

Cell viability studies demonstrated that most excipients utilized in the microcapsule formulations were well tolerated by both Caco-2 (Figure 8a) and MCF-7 (Figure 8b) cells at concentrations up to 1% (*w*/*v*), with viability values typically exceeding 80% relative to PBS control. Only Triton X-100 produced severe cytotoxic effects, as indicated by markedly reduced cell viability even at low concentrations, which aligns with its known detergent properties and established use as a positive control for cell lysis. Capryol^®^ 90, Labrasol^®^, and Transcutol^®^ HP exhibited a moderate, concentration-dependent decrease in cell viability, but values generally remained above 60% at concentrations up to 0.5% (*w*/*v*). In contrast, sodium alginate, PVP, and calcium chloride showed minimal cytotoxicity, further supporting their suitability for oral pharmaceutical formulations.

**Figure 9:**
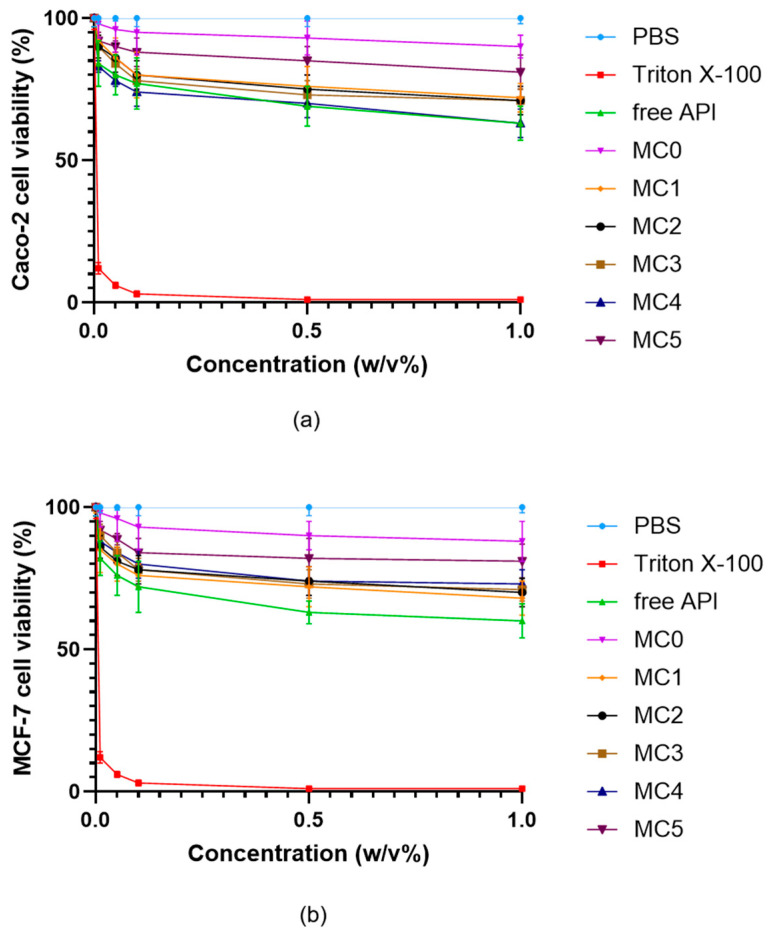
Viability of Caco-2 (**a**) and MCF-7 (**b**) cells incubated with the microcapsules in different concentrations. Cell viability is expressed as a percentage of the negative control (PBS). Data are presented as mean ± SD; *n* = 6. Two-way ANOVA followed by Dunnett’s test vs. the free active ingredient (free API) was performed to evaluate the effects of formulation type on cell viability. Significant differences (*p* < 0.01) were found for Triton X-100, PBS, MC5, and MC0 in either Caco-2 or MCF-7 cells.

Further assessment of the cytocompatibility was performed using all microcapsule formulations (MC0–MC5) and the free inhibitor molecule in both Caco-2 (Figure 9a) and MCF-7 (Figure 9b) cells across a range of concentrations up to 1% (*w*/*v*). All microcapsule formulations demonstrated high cell viability, typically remaining above 80% even at the highest tested concentrations. In contrast, the free API caused a more pronounced, concentration-dependent decrease in cell viability, particularly observable at higher concentrations (<80%). The results indicate that microencapsulation substantially reduces the cytotoxic effects of the ABCG2 inhibitor **7b** on both cell lines. This protective effect is most apparent for MC5 and MC4, which maintained the highest levels of viability, highlighting the biocompatibility of the SNEDDS-containing bead systems.

The results also show that MCF-7 cells exhibited a slightly greater reduction in viability at increasing concentrations compared to Caco-2 cells; however, this difference was not statistically significant. This indicates that, while MCF-7 cells may be somewhat more sensitive to the tested compounds.

### 3.9. Evaluation of Transepithelial Electrical Resistance

**Figure 10:**
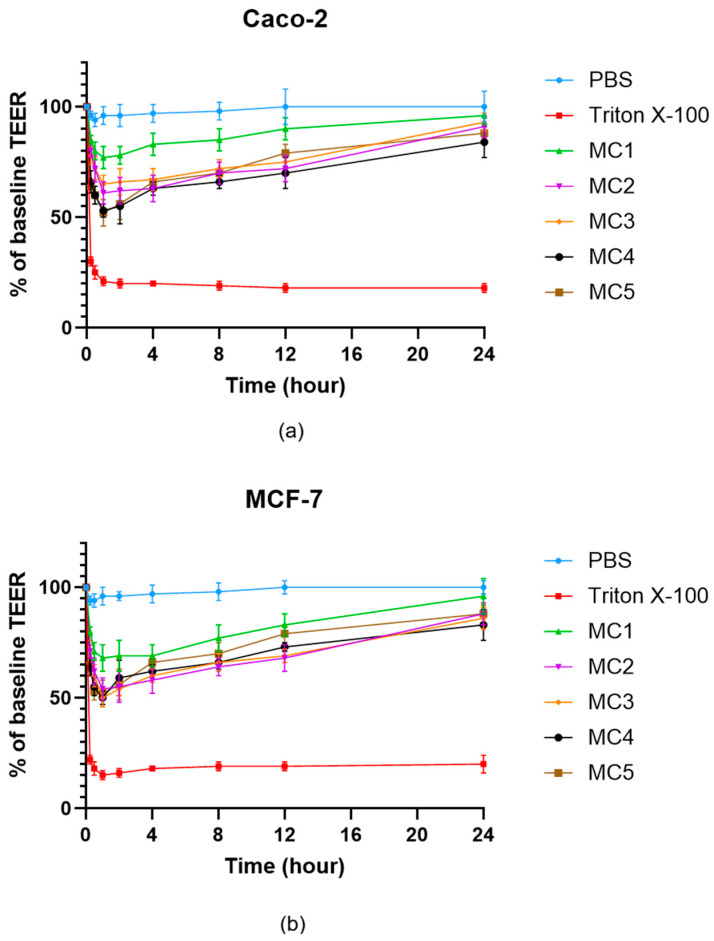
TEER measurement of Caco-2 (**a**) and MCF-7 (**b**) cell barrier integrity after treatment with microcapsules. Each data point represents the mean ± SD; *n* = 6. Data were analyzed by one-way ANOVA at the final time point, followed by Dunnett’s multiple comparisons test versus PBS. Significant differences (*p* < 0.05) were found for Triton X-100 and MC4 in either Caco-2 or MCF-7 cells.

Transepithelial electrical resistance (TEER) measurements were used to assess the effects of microcapsule formulations on the integrity of Caco-2 (Figure 10a) and MCF-7 (Figure 10b) cell monolayers over 24 h. Treatment with PBS maintained baseline TEER values in both cell types, confirming barrier integrity. In contrast, exposure to Triton X-100 led to a profound and sustained reduction in TEER, reflecting rapid disruption of cell junctions and barrier function. All microcapsule-treated groups (MC1–MC5) showed an initial decrease in TEER, followed by a gradual recovery over time, indicating that the formulations caused only a transient and reversible compromise of the epithelial barrier. By 24 h, TEER values for all microcapsule groups approached 80–90% of the original baseline in both Caco-2 and MCF-7 cells, suggesting restoration of monolayer integrity. No substantial differences were observed between the two cell lines, and all microcapsule formulations demonstrated a similar and acceptable safety profile with respect to epithelial barrier disruption.

### 3.10. Permeability Assay

**Figure 11:**
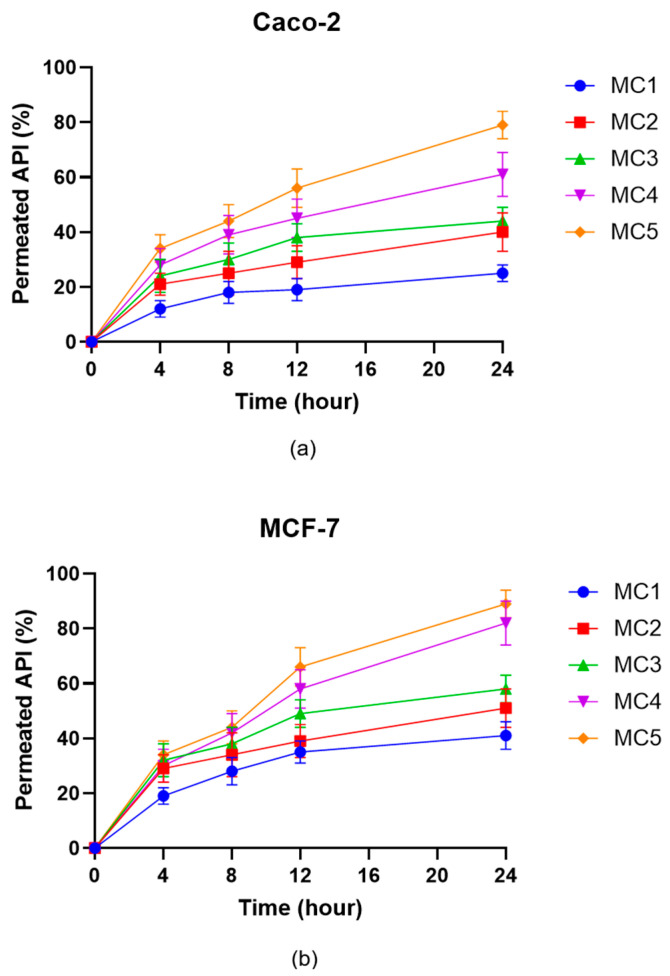
In vitro permeation of ABCG2 inhibitor **7b** (referred as API) from the microcapsules through Caco-2 (**a**) and MCF-7 (**b**) cells. Data are presented as mean ± SD, *n* = 6. Data were analyzed by one-way ANOVA at the final time point, followed by Dunnett’s multiple comparisons test versus MC1. In case of the Caco-2 cell line significant differences (*p* < 0.01) were found for MC2, MC3, MC4 and MC5, while for the MCF-7 cell line significant differences were observed for MC3, MC4 and MC5.

Permeation studies across Caco-2 (Figure 11a) and MCF-7 (Figure 11b) cell monolayers revealed marked differences among the microcapsule formulations with respect to the transport of the ABCG2 inhibitor **7b**. For both cell lines, the percentage of permeated API increased progressively over 24 h, with MC5 producing the highest permeation rates at all measured time points. MC4 also showed enhanced transcellular permeability relative to MC1–MC3, mirroring trends observed in the dissolution and enzymatic stability assays. In contrast, MC1 and MC2 consistently resulted in the lowest API permeation. While the absolute permeated amounts were higher in the MCF-7 model, the overall ranking of the microcapsule formulations in terms of API transport was preserved between the two cell lines. These findings indicate that SNEDDS-containing formulations (MC4 and MC5) efficiently promote transepithelial delivery of the ABCG2 inhibitor **7b**, which could translate into improved in vivo absorption following oral administration.

## 4. Discussion

The present study systematically evaluated the impact of SNEDDS formulation and microencapsulation strategies on the physicochemical properties, stability, release, biocompatibility, and permeability of the ABCG2 inhibitor **7b**. The ABCG2 efflux transporter is known to limit intestinal absorption of many drugs, and its inhibition represents a promising strategy for overcoming multidrug resistance and enhancing bioavailability of co-administered therapeutics [38]. However, many small-molecule ABCG2 inhibitors suffer from poor aqueous solubility and chemical instability, which complicate their oral delivery [39]. In this context, the combination of polymeric encapsulation and SNEDDS provides a versatile platform for protecting sensitive molecules and modulating their release behavior [40].

The findings demonstrate that careful selection and combination of surfactant and co-surfactant components, for example, the synergistic pairing of Labrasol^®^ and Transcutol^®^ HP, represent an effective means of maximizing emulsification efficiency, as evidenced by the marked increase in transmittance values when these were applied together. Similar enhancement in emulsification and solubilization capacity through surfactant–co-surfactant combinations has been reported by Patel J. et al., highlighting the critical role of excipient selection in SNEDDS optimization [28]. This improvement in emulsification directly correlates with superior drug solubilization capacity, a critical parameter for achieving high bioavailability of poorly water-soluble drug molecules, as also emphasized by Date A. A. and Patel V. F. in their work on lipid-based delivery systems [13,41].

Solubility studies established Transcutol^®^ HP and Capryol^®^ 90 as the most promising SNEDDS components, with significantly greater solubilizing capacities compared to Labrasol^®^ alone. These findings are in agreement with Kommuru et al., who demonstrated that medium-chain co-surfactants like Transcutol^®^ HP markedly improve the solubilization of hydrophobic APIs in SNEDDS [42]. These components not only enable increased drug loading but also contribute to the formation of stable nanoemulsions with narrow size distributions and robust negative zeta potential when combined. Drug loading efficiency remained consistently high in the optimized SNEDDS formulations, supporting the suitability of this approach for delivering hydrophobic APIs. The observed increase in droplet size upon API incorporation is consistent with previous reports by Shahba et al., reflecting the effective encapsulation of drug molecules within the nanoemulsion structure [43].

We aimed to develop and evaluate alginate-based microcapsule formulations for the encapsulation and oral delivery of the ABCG2 inhibitor **7b**. Various formulation strategies were explored to enhance the solubility, stability, and bioavailability of the compound by incorporating solubilizing excipients such as Transcutol^®^ HP, polyvinylpyrrolidone (PVP), and SNEDDS. The findings demonstrate that both the choice and combination of excipients play a crucial role in modulating the physicochemical and biopharmaceutical properties of the microcapsules.

The morphological differences observed among the formulations can be attributed to the influence of nonionic surfactants and solubilizers on the gelation and drying dynamics of alginate systems. In the control microcapsules, the absence of surfactants allowed for uniform ionic cross-linking between sodium alginate and calcium ions, resulting in smooth, spherical beads with high structural integrity. However, formulations containing Transcutol^®^ HP and SNEDDS components displayed rougher and less uniform surfaces. This effect likely arises from the amphiphilic nature of these excipients, which can disturb the homogeneity of the polymeric network and promote droplet coalescence during emulsification. Nonionic surfactants lower the interfacial tension between aqueous and organic phases, facilitating the formation of smaller but less stable droplets that may merge during hardening, yielding irregular morphologies.

Similar findings have been reported for alginate and lipid-based microencapsulation systems where surfactants such as Tween 80, Labrasol^®^, or Transcutol^®^ HP disrupted polymer homogeneity and resulted in rough or collapsed microcapsules [44,45,46].

Encapsulation efficiency analysis revealed a pronounced dependence on the inclusion of solubilizing and polymeric excipients, with MC5 (containing both SNEDDS and PVP) achieving the highest efficiency. The simplest system (MC1), containing only alginate, PVP, and the ABCG2 inhibitor **7b**, exhibited limited entrapment, likely due to inadequate solubilization of the hydrophobic API. The addition of Transcutol^®^ HP, a well-known solubilizer and penetration enhancer, significantly increased EE, consistent with previous studies reporting improved encapsulation of poorly soluble drugs using glycol-based co-solvents [47,48]. Formulations containing both Transcutol^®^ HP and PVP (MC3) exhibited markedly higher EE values compared to the basic alginate–PVP system (MC1), indicating improved solubilization and drug–polymer interactions. The most pronounced improvement was observed in SNEDDS-containing formulations (MC4 and MC5), in which the drug was pre-dissolved in a nanoemulsion phase before entrapment. Similar results have been described for hydrophobic APIs such as cannabidiol [49], curcumin [29,50] and cyclosporine [51], where SNEDDS pre-loading enhanced the solubility thus the bioavailability of the drug. This finding highlights the synergistic effect of multi-component systems for maximizing drug entrapment within the polymer matrix. Similar observations were made by El-Say and Peltonen et al., who reported increased entrapment efficiency upon co-formulation with hydrophilic polymers, which improve matrix hydration and drug distribution [52,53].

Enhanced swelling is beneficial for controlled release purposes, as it supports a higher degree of matrix hydration, more efficient diffusion, and ultimately, greater release of the encapsulated compound [54]. Our swelling studies further supported the influence of formulation components on the structural and hydration behavior of microcapsules. The alginate-only control (MC0) displayed the lowest swelling, while PVP- and SNEDDS-containing systems (particularly MC5) showed the highest equilibrium water uptake. This observation aligns with literature indicating that PVP increases the hydrophilicity and porosity of alginate matrices, facilitating fluid penetration and diffusion [55,56]. Overall, swelling behavior was positively influenced by the addition of hydrophilic and amphiphilic agents, which increased water uptake and contributed to a more porous and hydrated microsphere structure. Similar swelling-enhanced release mechanisms have been documented by Tiwari et al. in hydrogel- and alginate-based delivery systems [57]. The results suggest that optimized water uptake supports balanced structural flexibility and permeability, which are critical parameters for oral delivery vehicles [58].

The enzymatic stability assays clearly demonstrated the protective effect of microencapsulation, particularly for SNEDDS-loaded formulations (MC4 and MC5), which markedly outperformed their simpler counterparts and free drug in both gastric and intestinal conditions. The free ABCG2 inhibitor was highly susceptible to enzymatic and pH-induced degradation, whereas encapsulated formulations significantly prolonged stability in both simulated gastric and intestinal fluids. The superior protection afforded by MC4 and MC5 is attributed to the nanoemulsion phase of the SNEDDS, which acts as a physical barrier, reducing exposure to enzymatic attack and aqueous degradation. The nanoemulsion droplets formed a protective interfacial barrier, minimizing the contact between the API and enzymatic fluids. These results align with the findings of other studies, that similarly observed improved gastrointestinal stability of sensitive molecules through encapsulation in lipid-polymer hybrid systems [59,60]. This underscores the role of SNEDDS in shielding drug molecules from premature enzymatic degradation, which is a prerequisite for effective oral administration of biologics and labile APIs. The enhanced stability of MC4 and MC5 is especially relevant for oral administration, where enzymatic degradation is a major factor limiting the bioavailability of many molecule [61].

Dissolution and permeation studies further confirmed the advantages of SNEDDS-containing microcapsules as MC4 and MC5 not only delivered more rapid and extensive dissolution of the drug but also promoted higher transepithelial transport across both Caco-2 and MCF-7 monolayers. The improvement in permeability is likely attributable to the nanoemulsion-mediated increase in effective surface area and the solubilizing microenvironment provided by SNEDDS, facilitating API translocation across epithelial barriers. This observation is in good agreement with Porter et al., who demonstrated enhanced intestinal absorption of lipophilic drugs through SNEDDS-mediated permeability enhancement [62].

From a safety perspective, cytocompatibility assays indicated excellent tolerance for most excipients as well as for the microcapsule formulations, with only minimal, concentration-dependent reductions in viability and reversible effects on barrier integrity as reflected in TEER assays. Importantly, encapsulation significantly reduced the cytotoxicity of the free ABCG2 inhibitor, reflecting the protective effect of the polymeric and nanoemulsion matrices. This reduction in cellular toxicity is particularly advantageous for drug candidates with narrow therapeutic windows or potential off-target effects. The full recovery of TEER values after 24 h indicates that the microcapsules do not cause lasting disruption of cell junctions, confirming their compatibility with epithelial cell monolayers. These findings are consistent with the minimal cytotoxicity observed in cell viability assays. Notably, cell viability in MCF-7 cells was somewhat more sensitive to exposure, but differences between the two models were not statistically significant, underscoring the broad biocompatibility of the developed systems. These results agree with previous reports that described low cytotoxicity profiles for Labrasol^®^-, Transcutol^®^ HP-, and alginate-based formulations [63,64,65].

In summary, this study demonstrates that combining SNEDDS-based encapsulation with hydrophilic polymeric additives markedly enhances drug entrapment, stability, release, and permeability, while maintaining cellular compatibility. Despite the described morphological variations, the surfactant-containing formulations generally exhibit improved encapsulation efficiency, drug dispersion, and controlled release, suggesting that minor loss in morphological uniformity is compensated by enhanced functional performance. These results support the potential of this integrated approach for improving the oral delivery and therapeutic performance of challenging, poorly soluble or instable pharmaceutical agents, in line with the conclusions drawn by several other research groups working with advanced lipid-based formulations [48,51]. The optimized formulation successfully addressed key barriers to oral absorption, poor solubility, enzymatic degradation, and limited permeability, while maintaining excellent biocompatibility.

Such microcapsule systems hold promises for improving the oral bioavailability of efflux transporter inhibitors and other poorly soluble compounds. Importantly, the observed improvements in stability and permeability could contribute to more consistent pharmacokinetic profiles and potentially lower required doses, minimizing systemic toxicity risks. However, the inherent physicochemical liabilities of **7b** may still influence its post-absorption pharmacokinetics and restrict its ability to achieve therapeutically relevant concentrations in certain tissues, particularly poorly vascularized solid tumors. Future work should therefore not only include in vivo pharmacokinetic and pharmacodynamic studies to validate these in vitro observations but also assess whether the physicochemical profile of **7b** imposes constraints on tumor penetration and overall in vivo effectiveness. In addition, the use of ABCG2 inhibitor-loaded microcapsules could be further investigated as co-delivery systems to enhance the absorption of co-administered drugs subject to ABCG2-mediated efflux. Given that ABCG2 is widely expressed in the gastrointestinal tract [66], such systems could play a significant role in overcoming drug resistance and improving the oral delivery of targeted therapeutics.

## 5. Conclusions

In conclusion, this study clearly demonstrates that integrating SNEDDS technology with microencapsulation strategies provides a highly effective approach for improving the oral delivery of ABCG2 inhibitor **7b**. The careful optimization of excipient selection was shown to significantly enhance emulsification, stability, and solubilization potential, directly translating into improved drug loading and bioavailability for the poorly soluble inhibitor **7b**. High encapsulation efficiency and controlled swelling with hydrophilic and amphiphilic additives further enabled sustained release and superior protection against enzymatic degradation, while maintaining overall biocompatibility and cell viability. Notably, SNEDDS-containing microcapsules facilitated faster dissolution and higher transepithelial permeability across relevant cell models, thus supporting efficient intestinal absorption.

Taken together, these findings underscore the value of combining lipid-based delivery technologies and polymeric encapsulation to address the challenges associated with oral administration of hydrophobic and labile compounds. This integrated formulation approach can pave the way for more effective therapeutic exploitation of ABCG2 inhibitors and may be broadly applicable to other drugs with limited water solubility in the GI environment. Future studies should focus on in vivo pharmacokinetic and pharmacodynamic evaluations to confirm enhanced absorption and therapeutic performance observed in vitro.

## Figures and Tables

**Table 1:** Ingredients in the initial solutions used for microcapsule preparation.

Ingredients of Initial Solutions	MC0	MC1	MC2	MC3	MC4	MC5
Sodium alginate (1.5% *w*/*v*)	x	x	x	x	x	x
ABCG2 inhibitor **7b** (1% *w*/*v*)		x	x	x	x	x
Transcutol^®^ HP (0.1% *w*/*v*)			x	x		
PVP (2% *w*/*v*)		x		x		x
SNEDDS (5% *w*/*v*)					x	x

**Table 2:** Physicochemical characteristics of blank and ABCG2 inhibitor **7b** (API)-loaded SNEDDS formulations, including droplet size, polydispersity index (PDI), zeta potential, and drug loading efficiency. Each data point represents the mean ± SD; *n* = 6.

Composition	Droplet Size (nm)	PDI	Zeta Potential (mV)	Drug Loading Efficiency (%)
SNEDDS without API	45.313 ± 1.726	0.189 ± 0.009	−34.234 ± 1.884	
SNEDDS with API	124.872 ± 12.445	0.195 ± 0.011	−32.898 ± 2.012	98.066 ± 4.145

**Table 3:** Results of encapsulation efficiency measurements regarding the different formulations containing ABCG2 inhibitor **7b**.

Formulation	EE %
MC1	61.21 ± 3.88
MC2	72.33 ± 4.17
MC3	83.13 ± 3.02
MC4	91.04 ± 3.99
MC5	95.66 ± 2.86

## Data Availability

The data that support the findings of this study are available from the corresponding author (jozsa.liza@euipar.unideb.hu) with the permission of the head of the department, upon reasonable request.
